# Quantitative analysis of biofluid spots by coated blade spray mass spectrometry, a new approach to rapid screening

**DOI:** 10.1038/s41598-017-16494-z

**Published:** 2017-11-23

**Authors:** Germán Augusto Gómez-Ríos, Marcos Tascon, Nathaly Reyes-Garcés, Ezel Boyacı, Justen Poole, Janusz Pawliszyn

**Affiliations:** Department of Chemistry, University of Waterloo, Waterloo, Ontario, N2L 3G1 Canada

## Abstract

This study demonstrates the quantitative capabilities of coated blade spray (CBS) mass spectrometry (MS) for the concomitant analysis of multiple target substances in biofluid spots. In CBS-MS the analytes present in a given sample are first isolated and enriched in the thin coating of the CBS device. After a quick rinsing of the blade surface, as to remove remaining matrix, the analytes are quickly desorbed with the help of a solvent and then directly electrosprayed into the MS analyzer. Diverse pain management drugs, controlled substances, and therapeutic medications were successfully determined using only 10 µL of biofluid, with limits of quantitation in the low/sub ng·mL^−1^ level attained within 7 minutes.

## Introduction

**Figure 1:**
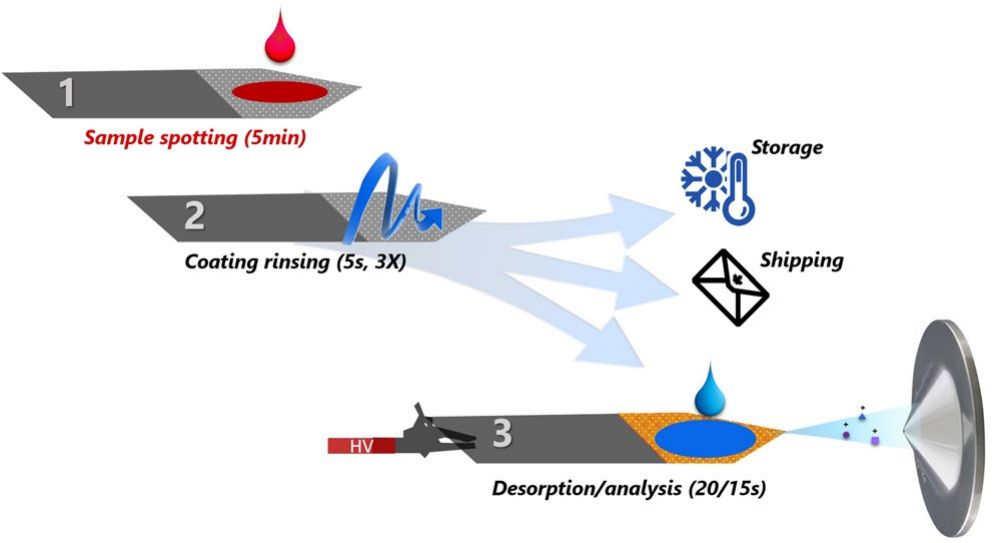
Experimental set-up for quantitative analysis of blood or plasma droplets via Coated Blade Spray-Mass Spectrometry (CBS-MS).

**Figure 2:**
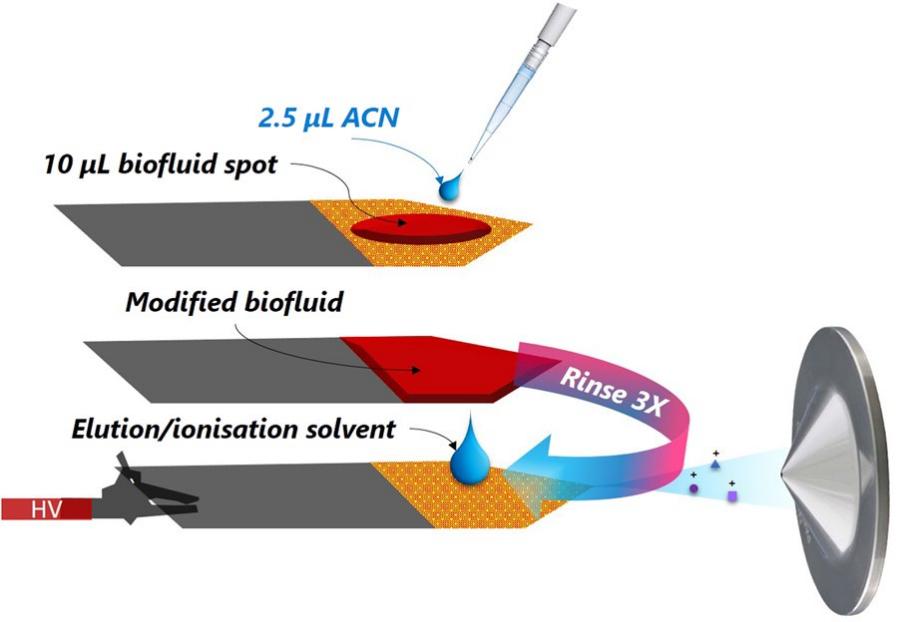
SPME-CAN methodology towards analysis of target compounds heavily bound to proteins and/or red blood cells via CBS.

Efficient, simple, and cost-effective methods that allow for quantitative analysis of small volumes of biofluids are critical for the advancement of personalized medicine and drug development. State-of-the-art mass spectrometry (MS) instrumentation in combination with innovative and easy-to-use microsampling technologies have facilitated the development of new analytical methodologies^1,2^. Applications such as new-born screening^3,4^, therapeutic drug monitoring^2,5–7^, and drug metabolism pharmacokinetics (DMPK)^8^ have greatly benefitted from these advances. Such microsampling devices are consisted of, or contain, a piece of paper or polymeric absorbent in which a droplet of biofluid can be collected then dried. Following, devices can be either stored for further studies or sent to the laboratory for immediate analysis. Typically, the analytical workflow for determination of analytes of interest collected on these devices consists of multiple steps prior to quantitation via MS, including liquid extraction, extract clean-up, analyte elution (e.g. by using solid phase extraction, SPE), and chromatographic separation^9^. Aiming to increase the throughput of analysis, on-line technologies that combine all these steps have been developed and thoroughly assessed by other researchers^10^. Further, in the last ten years, scientists have developed multiple ground-breaking technologies allowing for the eradication of the sample-prep/separation stages from the analytical work-flow^1^. These technologies, allowing for direct MS analysis of dried biofluid spots (e.g. paper spray, PS), have gained remarkable popularity, being adapted in numerous applications^1,11^. However, sample preparation cannot be entirely overlooked; indeed, several unavoidable effects intrinsic to ionization and detection processes can resultantly emerge from a lack of sufficient sample preparation, including ion suppression, poor sensitivity, and potential instrument contamination^12,13^. Aiming to solve these issues, a new chapter in this novel era of MS was written by merging micro-sample preparation technologies with ambient ionization MS approaches^2,14,15^. However, while such methods can certainly address the aforementioned effects, not all of them provide a pragmatic approach to analysis (*e.g*. complex operation, long analysis times, or expensive equipment/parts required per analysis). In this regard, there still exists a demand for a tool that not only improves limits of quantitation (LOQ) and minimizes matrix effects, but that can also offer high throughput compatibility for rapid diagnostics^16^. Coated Blade Spray (CBS), an SPME-based technology designed for enrichment of analytes of interest from complex sample matrices, can be directly coupled with MS instruments for rapid quantitative or qualitative analysis^17^. CBS can be described as a sword-like stainless steel sheet coated with polymeric adsorbent particles, which act as a solid-substrate ESI source (Fig. 1)^18,19^. Unlike classical microsampling devices or PS, CBS functions by extracting/enriching analytes of interest from a given sample, rather than through a collection of dried sample spots^20^. Certainly, extracting analytes on a selective adsorbent—rather than collecting analyte-matrix on a non-selective substrate—is more advantageous because it safeguards against the presence of other matrix components, such as enzymes, that can facilitate the conversion/degradation of the target compound. When compare to PS, CBS offers additional advantages such better electrical conductivity, well-defined sharp tip and no-tendency to become deformed when handling large solvent/sample volumes. Contrary to general assumptions^21–23^, CBS has been shown to handle a broad range of sample volumes (*i.e*. µL to L)^17,20,24^. Herein, having accepted the challenge of employing CBS towards analysis of minimum amounts of sample (≤10 µL), we present a thorough validation of the quantitation capabilities of CBS for analysis of broad range of analytes in small volumes of biofluids. Aiming to reach lower limits of quantitation, a novel methodology that defies the solventless phylosophy of SPME-based technologies is also disclosed (Fig. 2). This workflow, named SPME-CAN (pun intended), demonstrates that SPME-based technologies not only CAN quantify multiple target analytes in small sample volumes, but also without a pre-conditioning step and with better performance than other substrate-spray technologies without a pre-concentration step^14,21^.

## Experimental Section

### Chemical, reagents and materials

Formic acid was purchased from Sigma-Aldrich (Saint Louis, USA), and LC-MS-grade methanol (MeOH), acetonitrile (ACN), isopropanol (IPA) and water were purchased from Fisher Scientific. The following compounds were selected as model analytes for evaluating the quantitation capabilities of CBS in droplet analysis: methamphetamine, methamphetamine-d5, carbamazepine, carbamazepine-d10, propranolol, propranolol-d7, clenbuterol, clebuterol-d9, diazepam, diazepam-d5, codeine, codeine-d3, cocaine, cocaine-d3, sertraline, sertraline-d3, citalopram, citalopram-d6, fentanyl, fentanyl-d5, buprenorphine, buprenorphine-d4, morphine, morphine-d6, methadone, methadone-d3, oxycodone, lorazepam, bisoprolol and stanozolol. All standards were acquired from Cerilliant Corporation (Round Rock, TX, USA). As noted, deuterated analogues of most analytes were used to correct for intra- and inter-experiment variability. For further details regarding compound properties, and selected reaction monitoring (SRM) transitions, see Table S1. Individual stock standard solutions were prepared in methanol at a concentration of 1000 µg·mL-1 and stored at −80 °C. Stainless steel blades, which were purchased from Shimifrez Inc. (Concord, Ontario, Canada), were then coated using a slurry of hydrophilic lipophilic balance particles and polyacrylonitrile (HLB-PAN) according to a protocol developed in our laboratory (see supplementary information material for further details) with HLB particles kindly provided by Waters Corporation. The coating length and thickness was 15 mm and 10 µm, respectively.

### Biological samples

A phosphate-buffered saline solution (PBS) (pH 7.4) was prepared by adding 8.0 g of sodium chloride, 0.2 g of potassium chloride, 0.2 g of potassium phosphate, and 1.44 g of sodium phosphate to 1 L of nanopure water. Human plasma (with potassium (K2) ethylenediaminetetraacetic acid (EDTA) as anticoagulant) had been pooled from different batches and was purchased from Bioreclamation IVT (Baltimore, MA, U.S.A). Pooled whole blood from healthy donors in potassium K2-EDTA was purchased from Bioreclamation IVT (Baltimore, MA, U.S.A.). All the plasma and blood samples were spiked and stored overnight at 4 °C in order to achieve the drug-protein binding equilibrium. All the experiments and methods herein reported were done with the approval of the University of Waterloo’s Office of Research Ethical Board.

### Mass Spectrometry

All the experiments described in this manuscript were carried out using a TSQ Quantiva mass spectrometer (Thermo Fisher Scientific, San Jose, California, USA), and data processing was performed using Trace Finder 3.3 (Thermo Fisher Scientific, San Jose, California, USA). To guarantee that the blades were accurately positioned in front of the mass spectrometer during all experiments, an in-house ionization source was built at the University of Waterloo. Once the CBS had been installed on the interface, 10 µL of a 95:5 MeOH/water v/v 0.1% formic acid solution was applied to the coated area in order to desorb the analytes (t ≤ 20s). After analyte elution on the desorption solution, a voltage of 4 kV was established between the CBS and the MS entrance to generate electrospray from the tip of the blade. All analyses were carried out in positive ionization mode. Optimum collision energy and RF-lens conditions were tuned for each compound via the direct infusion of methanolic standards. MS/MS transitions, optimum collision energy (CE), and RF-lens voltages for each analyte can be found in Table S1.

### Analytical methodology

All the CBS devices were cleaned after manufacturing for 30 min using a 40:40:20 (MeOH/ACN/IPA, v/v/v) solution and then conditioned for 30 min with a 50:50 (MeOH/water, v/v) solution. The main objective of the cleaning and preconditiong steps is to remove any byproducts of the manufacuring process (e.g. monomers, glue, porogones). It is also important to point-out that, regardless of the application, HLB-coated CBS can be dried prior to the extraction step. As illustrated in Fig. 1, the analytical workflow consists of three simple steps. First, 10 µL of biofluid is spotted onto the coated area of a dried blade, then left to interact with the extracting particles for 5 minutes. Next, the CBS is rapidly rinsed for 10 seconds with water, aiming to remove any potential matrix that could have adhered to the surface. Subsequently, the blade is placed in front of the MS system for analysis, and a droplet (10 µL) of elution/spraying solvent is added onto the coated area. Finally, after 20 seconds, ions of the extracted/pre-concentrated analytes are generated by applying a high electric field (+4 kV) between the blade and the mass spectrometer^17^. All the methods herein reported were performed according to University of Waterloo safety guidelines and regulations.

### Method Validation

The methodologies were validated with respect to linearity, precision, accuracy, and LOQ. Calibration functions were constructed on the basis of the signal ratio of the analyte and its isotopologue (A/Is) for 10 concertation levels in three independent replicates from 0.25 ng·mL^−1^ to 100 ng·mL^−1^. Furthermore, three validation points at concentrations of 3, 40, and 80 ng·mL^−1^ were analyzed in order to assess precision and accuracy. LOQs were calculated as the lowest calibration point with precision values lower than 20%.

## Results and Discussion

An initial assessment of CBS as a tool for analysis of biofluid spots was performed by employing, as model, phosphate buffered saline (PBS) samples spiked with 17 compounds from different classes and comprising a broad range of molecular weights, functional groups, and polarities (Table S1, ESI†), including controlled substances (e.g. clenbuterol), pain management drugs (e.g. buprenorphine), and drugs of abuse (e.g. fentanyl). Validation of the method was carried out by employing the matrix-matched calibration approach, and calibration functions were constructed on the basis of the signal ratio of the analyte and the internal standard (A/Is) for ten points in four independent experiments, covering a range between 0.25 and 100 ng·mL^−1^. As shown in Table S2, by using blades coated with hydrophilic–lipophilic balanced (HLB) particles, LOQs equal or lower than 1 ng·mL^−1^ and outstanding figures of merit were reached for all analytes under study (*i.e*., SPME balance coverage: extraction/enrichment of compounds from a broad range of polarities). In view of these promising results, CBS was then employed towards analysis of analytes spiked on human plasma. As summarized in Table S3, LOQs for all compounds were, in almost all cases, below the minimum required performance levels (MRPL) set by the World Andi-Doping Agency (WADA), the cut-off established by the Substance Abuse and Mental Health Services Administration (SAMHSA), or the analytical quantitation limits established by certified clinical laboratories (i.e., LOQ ~ 1–5 ng·mL^−1^). As shown in Table S4, similar results were obtained for analyses of blood samples spiked with the same target analytes (i.e., LOQ ~ 1–10 ng·mL^−1^). Although LOQs were higher for plasma and blood spots, the validation of the methodology yielded reassuring results at all concentration levels (Tables S2-4, ESI†). Given that CBS, like any other SPME device, extracts via free concentration, analytes largely bound to plasma proteins or red blood cells are expected to provide lower extraction recoveries in comparison to those provided by PBS (*i.e*., worst-case scenario for SPME-related technologies)^25^. Aware of the intrinsic limitations of CBS, we decided to shift the paradigm and defy the solventless philosophy of SPME^26^.

**Figure 3:**
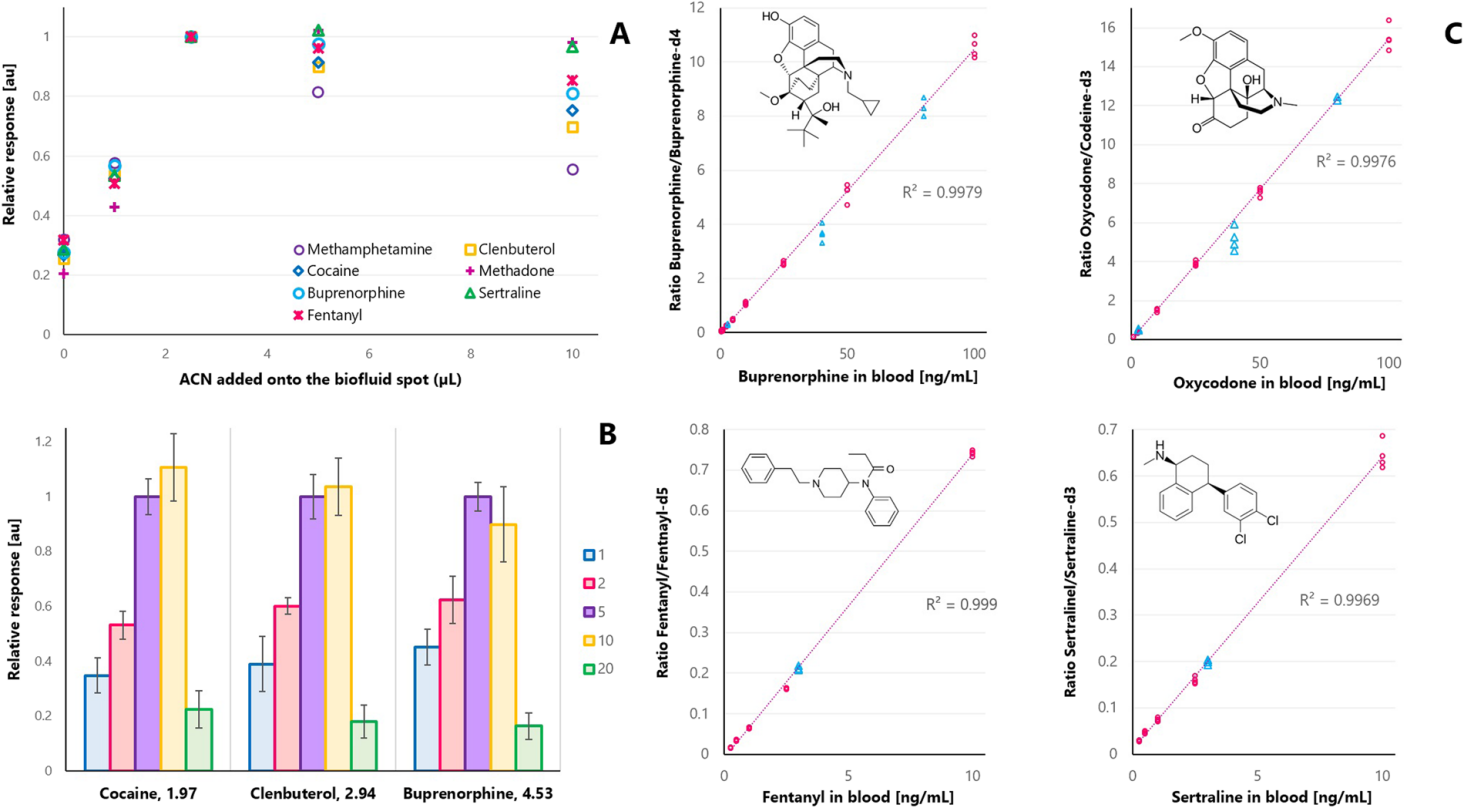
(**A**) Optimization of the volume of ACN added to biofluid spot. (**B**) Optimization of interaction time (min) between modified spot and coated blade (**C)**. Quantitative analysis of whole blood spiked with buprenorphine (0.5–100 ng mL^−1^), oxycodone (2.5–100 ng mL^−1^), fentanyl (0.1 ng mL^−1^ to 10 ng mL^−1^), and sertraline (0.25–10 ng mL^−1^). Total sample volume was 10 µL and total analysis time ≤7 min via MS/MS.

As portrayed in Fig. 2, our idea entailed adding a minuscule amount of organic solvent (*i.e*. acetonitrile, ACN) to the biofluid spot so as to modify the matrix viscosity as well as the analyte-protein-binding properties, resultantly increasing the free concentration of the analytes under study, and thus facilitating their extraction onto the coating particles^23^. However, the addition of solvent onto an SPME surface holding a biofluid droplet brings up extra challenges for analyte quantitation. For instance, if the amount of organic solvent on the coating is too large, analyte partition may be driven onto the solvent layer rather than onto the extractive particles, due to a dramatic decrease in the analyte partition coefficient (K_fs_), and consequently, decrease the free concentration of the analyte. Likewise, if extraction/enrichment time is too long (t ≥ 5 min), precipitation of macromolecules (*i.e*., proteins^6^), as well as blood skeletonization on the coating, may occur. Such events might lead to significant ionization suppression and potential instrument contamination. Thus, optimization of solvent volume and interaction time were critical steps in the development of this method. As shown in Fig. 3A,B, the highest response was attained when using 2.5 µL of ACN and 5 minutes of contact time. Unquestionably, these results evidence the relevance of the clean-up feature intrinsic of SPME-based devices such CBS, which are inbuilt so as to prevent the undesired attachment of potential contaminants/interferences. As can be seen in Fig. 3C and Table 1, employment of this new method, named SPME-CAN, resulted in lower LOQs for all studied probes (i.e., 2–20 fold enhancement), without sacrificing total analysis time. It is important to point out that deuterated internal standards were not available for all analytes under investigation (salbutamol, oxycodone, bisoprolol, lorazepam). Nonetheless, the presented findings demonstrated that CBS-MS/MS was a suitable technique for quantitative analysis of all the studied compounds, even when the deuterated analogue of the target compound was not available (see Fig. 3C).

**Table 1:** Figures of merit for determination of multiple substances in blood spots by SPME-CAN methodology via CBS-MS/MS.

Compound	Log P^a^	Protein binding (%)^b^	LOQ,	LOQ,	Accuracy (n = 4), %			Precision (n = 4), %		
			Spot-alone	SPME-CAN	3 ng/mL	40 ng/mL	80 ng/mL	3 ng/mL	40 ng/mL	80 ng/mL
			ng/mL	ng/mL						
Salbutamol	0.44	—	10	2.5	113.6	81.7	86.3	7.1	7.5	12.7
Morphine	0.99	30–40	10	2.5	118.6	86.9	87.2	6.5	6.3	3.6
Oxycodone	1.04	45	10	2.5	110.7	81.9	93.6	6.9	11.4	5.6
Codeine	1.20	7–25	2.5	0.5	94.2	80.8	93.7	6.5	4	3.2
Cocaine	1.97	—	2.5	0.25	92.9	86.4	96.1	3.5	2.4	1.2
Methamphetamine	2.23	—	10	0.5	90.1	84.3	95.7	2.9	2.1	1.6
Bisoprolol	2.30	30	1	0.5	95.8	87.7	95.6	4.2	3.1	7.7
Diazepam	2.63	98.5	2.5	0.5	95.5	80.5	93.4	3.7	4.5	2.3
Carbamazepine	2.77	76	1	0.25	113.2	90.7	95.3	1.7	1.8	2.9
Clenbuterol	2.94	—	1	0.5	95.9	83.3	96.4	2.8	1.7	0.9
Lorazepam	2.98	89–93	10	2.5	128.4	91.5	103.6	8.5	8.7	16.4
Propranolol	3.03	>90	5	0.5	92.0	83.2	92.6	1.8	1.9	1.9
Citalopram	3.58	80	2.5	0.5	95.7	85.3	94.6	4.5	2.5	2.4
Fentanyl	4.12	80–85	2.5	0.25	93.2	85.7	95.2	2.3	1.7	0.8
Methadone	4.14	85–90	1	0.25	96.2	92.8	93.5	2.6	1.5	0.9
Buprenorphine	4.53	96	1	0.5	98.4	85.8	97.8	7.2	8.3	4.1
Sertraline	5.06	98	5	0.25	96.5	87.1	95.1	2.4	8.5	3.3

^a,b^Log P and protein binding values were obtained from www.drugbank.ca.

**Figure 4:**
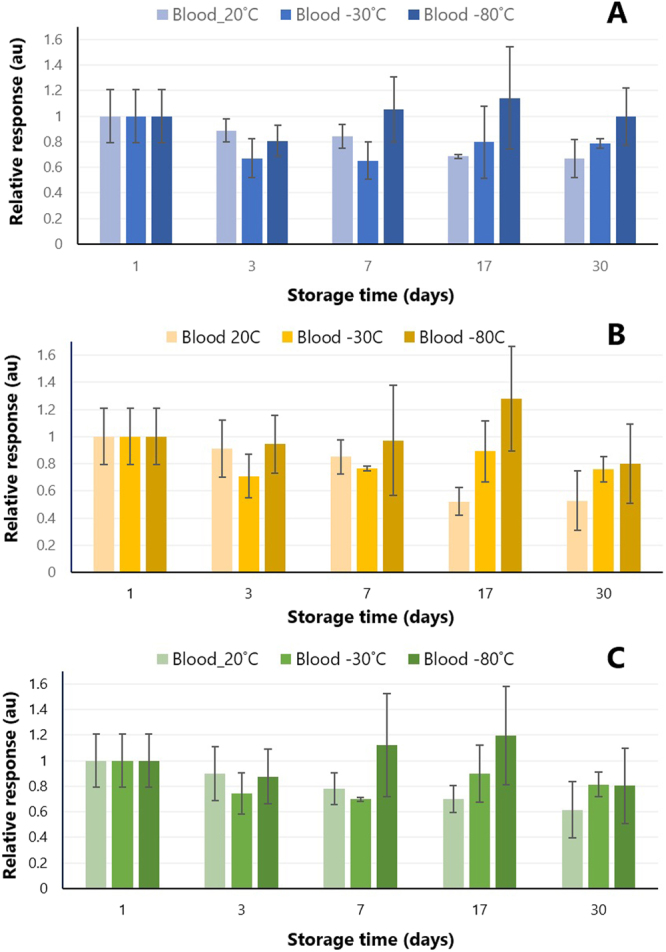
Storage stability of analytes extracted from blood spots on CBS devices for several days. (**A**) Cocaine; (**B**) Methamphetamine; (**C)** Fentanyl.

An additional feature of CBS for point-of-care applications is its ability to guarantee analyte stability on the coating prior to instrumental analysis. Our findings showed that the majority of the compounds were stable on the coating, even at room temperature, for up to 7 days (Fig. 4). Certainly, further stability can be accomplished by storing the blades at low temperatures (−30/−80 °C)^26,27^. While the stability of CBS has only been evaluated to 30 days at freezing conditions at the this time, a long-term storage evaluation experiment (>6 months) is currently under way in our laboratory.

## Conclusions

In summary, the potential of CBS for analysis of various compounds present in small volumes of biofluids was thoroughly validated. Unlike other SPME-MS approaches, no additional instrumentation is required for analysis, as the blade acts as both the extraction device and ionization-source^16,28^. Similar to dried blood spot (DBS) or PS methods, sample containers are not needed for sample collection, as the sample can be simply spotted onto the coated area of the blade. Thus, after enriching the analytes on the coating, the CBS device can be shipped to the laboratory for immediate analysis, or simply stored under cold chain pending examination (Fig. 1)^29,30^. Likewise, we foresee the application of CBS towards analysis of less invasive matrices such as urine or saliva^31,32^, as an extractive matrix spot^33,34^. In the same line, one could see the suitability of this technology for fingerprinting applications and population studies of microorganisms such as fungi and bacteria^35–37^. Certainly, the SPME-CAN methodology herein proposed can be easily implemented with other SPME substrates consisted of a flat geometry, such as SPME-Transmission Mode^38^, for spot analyses. As a future direction, our current work focuses on the development of a technology that allows for simple pre-loading of internal standards to facilitate quantitation of small sample volumes and a hassle-free sample manipulation approach^39,40^. Definitely, CBS-MS is not meant to solve all analytical problems, and combinations with on-line technologies such as differential mobility spectrometry^41^ (or ion-mobility^42^) and multiple reaction monitoring with multistage fragmentation (MRM^3^) may be necessary to quantify more challenging compounds (*e.g*. isobars with share fragment ions)^16^. Nevertheless, the findings herein presented are quite encouraging towards the development of a cost-effective tool that can be easily implemented for on-site analysis and rapid diagnostics^43–45^.

## Electronic supplementary material

Supplementary information

## References

[1] Ferreira CR (2015). Ambient Ionization Mass Spectrometry for Point-of-Care Diagnostics and Other Clinical Measurements. Clin. Chem..

[2] Gómez-Ríos GA, Reyes-Garcés N, Bojko B, Pawliszyn J (2016). Biocompatible Solid-Phase Microextraction Nanoelectrospray Ionization: An Unexploited Tool in Bioanalysis. Anal. Chem..

[3] Wilcken B, Wiley V, Hammond J, Carpenter K (2003). Screening Newborns for Inborn Errors of Metabolism by Tandem Mass Spectrometry. N. Engl. J. Med..

[4] Zytkovicz, T. H. *et al*. Tandem Mass Spectrometric Analysis for Amino, Organic, and Fatty Acid Disorders in Newborn Dried Blood Spots. *Clin. Chem*. **47** (2001). 11673361

[5] Verplaetse R, Henion J (2016). Hematocrit-Independent Quantitation of Stimulants in Dried Blood Spots: Pipet versus Microfluidic-Based Volumetric Sampling Coupled with Automated Flow-Through Desorption and Online Solid Phase Extraction-LC-MS/MS Bioanalysis. Anal. Chem..

[6] Ryona I, Henion J (2016). A Book-Type Dried Plasma Spot Card for Automated Flow-Through Elution Coupled with Online SPE-LC-MS/MS Bioanalysis of Opioids and Stimulants in blood. Anal. Chem..

[7] Manicke NE, Abu-Rabie P, Spooner N, Ouyang Z, Cooks RG (2011). Quantitative Analysis of Therapeutic Drugs in Dried Blood Spot Samples by Paper Spray Mass Spectrometry: An Avenue toTherapeutic Drug Monitoring. J. Am. Soc. Mass Spectrom..

[8] Rowland M, Emmons GT (2010). Use of dried blood spots in drug development: pharmacokinetic considerations. AAPS J..

[9] Denniff P, Spooner N (2014). Volumetric Absorptive Microsampling: A Dried Sample Collection Technique for Quantitative Bioanalysis. Anal. Chem..

[10] Oliveira RV, Henion J, Wickremsinhe E (2014). Fully-Automated Approach for Online Dried Blood Spot Extraction and Bioanalysis by Two-Dimensional-Liquid Chromatography Coupled with High-Resolution Quadrupole Time-of-Flight Mass Spectrometry. Anal. Chem..

[11] Wang H, Liu J, Graham Cooks R, Ouyang Z (2010). Paper spray for direct analysis of complex mixtures using mass spectrometry. Angew. Chemie.

[12] Fang L (2016). Coupling solid-phase microextraction with ambient mass spectrometry: Strategies and applications. TrAC - Trends Anal. Chem..

[13] Souza-Silva, É. A. *et al*. A critical review of the state of the art of solid-phase microextraction of complex matrices iii. bioanalytical and clinical applications. *TrAC Trends Anal. Chem*., 10.1016/j.trac.2015.04.017 (2015).

[14] Zhang C, Manicke NE (2015). Development of a Paper Spray Mass Spectrometry Cartridge with Integrated Solid Phase Extraction for Bioanalysis. Anal. Chem..

[15] Joshi, S., Zuilhof, H., van Beek, T. A. & Nielen, M. W. F. Biochip Spray: Simplified Coupling of Surface Plasmon Resonance Biosensing and Mass Spectrometry. *Anal. Chem*. acs.analchem.6b04012, 10.1021/acs.analchem.6b04012 (2017). 10.1021/acs.analchem.6b04012 PMC5348099 28208290

[16] Gómez-Ríos GA (2017). Open Port Probe Sampling Interface for the Direct Coupling of Biocompatible Solid-Phase Microextraction to Atmospheric Pressure Ionization Mass Spectrometry. Anal. Chem..

[17] Gómez-Ríos GA, Pawliszyn J (2014). Development of coated blade spray ionization mass spectrometry for the quantitation of target analytes present in complex matrices. Angew. Chemie.

[18] Venter AR, Douglass KA, Shelley JT, Hasman G, Honarvar E (2014). Mechanisms of real-time, proximal sample processing during ambient ionization mass spectrometry. Anal. Chem..

[19] Tascon M (2017). High-Throughput Screening and Quantitation of Target Compounds in Biofluids by Coated Blade Spray-Mass Spectrometry. Anal. Chem..

[20] Piri-Moghadam H (2016). Fast Quantitation of Target Analytes in Small Volumes of Complex Samples by Matrix-Compatible Solid-Phase Microextraction Devices. Angew. Chemie.

[21] Manicke NE, Bills BJ, Zhang C (2016). Analysis of biofluids by paper spray MS: advances and challenges. Bioanalysis.

[22] Bills BJ, Manicke NE (2016). Development of a prototype blood fractionation cartridge for plasma analysis by paper spray mass spectrometry. Clin. Mass Spectrom..

[23] Damon DE (2016). Direct Biofluid Analysis Using Hydrophobic Paper Spray Mass Spectrometry. Anal. Chem..

[24] Tascon, M. *et al*. Ultra-fast quantitation of voriconazole in human plasma by coated blade spray mass spectrometry. *J. Pharm. Biomed. Anal*., 10.1016/j.jpba.2017.03.009, (2017). 10.1016/j.jpba.2017.03.009 28318747

[25] Reyes-Garcés N, Bojko B, Pawliszyn J (2014). High throughput quantification of prohibited substances in plasma using thin film solid phase microextraction. J. Chromatogr. A.

[26] Ouyang G, Vuckovic D, Pawliszyn J (2011). Nondestructive sampling of living systems using *in vivo* solid-phase microextraction. Chem. Rev..

[27] Cudjoe E, Bojko B, de Lannoy I, Saldivia V, Pawliszyn J (2013). Solid-phase microextraction: a complementary *in vivo* sampling method to microdialysis. Angew. Chem. Int. Ed. Engl..

[28] Mirabelli MF, Wolf J-CC, Zenobi R (2016). Direct Coupling of Solid-Phase Microextraction with Mass Spectrometry: Sub-pg/g Sensitivity Achieved Using a Dielectric Barrier Discharge Ionization Source. Anal. Chem..

[29] Mei, J. In *Dried Blood Spots* 21–31 (John Wiley & Sons, Inc. doi:10.1002/9781118890837.ch3, 2014).

[30] McKenna, J. *et al*. Detection of Chemical Warfare Agent Simulants and Hydrolysis Products in Biological Samples by Paper Spray Mass Spectrometry. *Analyst*, 10.1039/C7AN00144D (2017). 10.1039/c7an00144d 28338135

[31] Reyes-Garcés N, Bojko B, Hein D, Pawliszyn J (2015). Solid phase microextraction devices prepared on plastic support as potential single-use samplers for bioanalytical applications. Anal. Chem..

[32] Numako M (2016). Dried Saliva Spot (DSS) as a Convenient and Reliable Sampling for Bioanalysis: An Application for the Diagnosis of Diabetes Mellitus. Anal. Chem..

[33] Déglon J, Leuthold LA, Thomas A (2015). Potential missing steps for a wide use of dried matrix spots in biomedical analysis. Bioanalysis.

[34] Pruski P (2017). Medical Swab Analysis Using Desorption Electrospray Ionization Mass Spectrometry: A Noninvasive Approach for Mucosal Diagnostics. Anal. Chem..

[35] Zhou Z, Lee JK, Kim SC, Zare RN (2016). Nanotip Ambient Ionization Mass Spectrometry. Anal. Chem.

[36] Yan C (2017). Real-Time Screening of Biocatalysts in Live Bacterial Colonies. J. Am. Chem. Soc..

[37] Golf O (2015). Rapid evaporative ionization mass spectrometry imaging platform for direct mapping from bulk tissue and bacterial growth media. Anal. Chem..

[38] Gómez-Ríos GAGA, Pawliszyn J (2014). Solid phase microextraction (SPME)-transmission mode (TM) pushes down detection limits in direct analysis in real time (DART). Chem. Commun..

[39] Yannell KE, Kesely KR, Chien HD, Kissinger CB, Cooks RG (2017). Comparison of paper spray mass spectrometry analysis of dried blood spots from devices used for in-field collection of clinical samples. Anal. Bioanal. Chem..

[40] Liu, J., Cooks, R. G. & Ouyang, Z. Enabling Quantitative Analysis in Ambient Ionization Mass Spectrometry: Internal Standard Coated Capillary Samplers. 5632–5636, 10.1021/ac401056q (2013). 10.1021/ac401056q PMC3705643 23731380

[41] Schneider BB, Nazarov EG, Londry F, Vouros P, Covey TR (2016). Differential mobility spectrometry/mass spectrometry history, theory, design optimization, simulations, and applications. Mass Spectrometry Reviews.

[42] Chouinard CD, Wei MS, Beekman CR, Kemperman RHJ, Yost RA (2016). Ion mobility in clinical analysis: Current progress and future perspectives. Clin. Chem..

[43] Bhamla MS (2017). Hand-powered ultralow-cost paper centrifuge. Nat. Biomed. Eng..

[44] Snyder DT, Pulliam CJ, Ouyang Z, Cooks RG (2016). Miniature and Fieldable Mass Spectrometers: Recent Advances. Anal. Chem..

[45] Thevis M, Geyer H, Tretzel L, Schänzer W (2016). Sports drug testing using complementary matrices: Advantages and limitations. J. Pharm. Biomed. Anal..

