# Supplementary material for: Quantitative analysis of biofluid spots by coated blade spray mass spectrometry, a new approach to rapid screening
Source: Sci Rep. 2017 Nov 23;7:16104. doi: 10.1038/s41598-017-16494-z (PMC5701014; doi:10.1038/s41598-017-16494-z)
Supplement: Supplementary file 1 — Supplementary information [file 41598_2017_16494_MOESM1_ESM.doc]

**Supporting Information**

**Scientific Reports**

**Quantitative analysis of biofluid spots by coated blade spray mass spectrometry, a new approach to rapid screening**

Germán Augusto Gómez-Ríos^†^, Marcos Tascon^†^, Nathaly Reyes-Garcés, Ezel Boyacı, Justen Poole, Janusz Pawliszyn*

*Department of Chemistry, University of Waterloo, Waterloo, Ontario N2L 3G1, Canada*

^†^These authors contributed equally

*Corresponding author:

Tel.: +1 519 888 4641; fax: +1 519 746 0435. E-mail address: janusz@uwaterloo.ca (J. P.).

**Manufacturing protocol for Coated Blade Spray devices**

1. **Coating slurry preparation**

The dipping solution is prepared by mixing 5 g of polyacrylonitrile (PAN) solution and 72.5 mL of dimethyl formamide (DMF) on a container. This mixture is heated at 90 °C for 1 hour. After cooling down, 6.3g of the solution are mixed with 0.65g of HLB particles (5 µm) on a 20 mL vial overnight prior to start the coating procedure.

1. **Coating manufacturing**

Prior to any coating application, CBS should be etched with hydrochloric acid (HCl, 37 %) for 15 minutes. After etching the devices must be thoroughly clean with water and acetone and they should be kept on a desiccator with nitrogen flow prior to execute the coating process. Ideally, the coating solution should be continuously stirred during the coating procedure. For coating, the desired coated area (e.g. 15 mm) is immersed onto the slurry solution for approximately 10 seconds and subsequently remove and cure at 125 °C for 1 min. For the preparation of CBS used in this manuscript a single dipping step was followed.

**Table S1** Target analytes, hydrophobicity (expressed as LogP), minimum required performance levels (MRPL, ng mL^-1^) or therapeutic range (TR, ng mL^-1^), and monitored SRM transitions for individual model compounds, measured in positive ionization mode with CBS-MS/MS. All the experiments were performed on a TSQ-Quantiva (Thermo Fisher Scientific, San Jose, California, USA)

| **#** | **Compound** | **LogP P** | **MRPL/TR*** | **Exact mass (m/z)** | **Precursor (m/z)** | **Product (m/z)** | **Collision Energy (V)** | **RF Lens (V)** |
| --- | --- | --- | --- | --- | --- | --- | --- | --- |
| 1 | Methamphetamine | 2.23 | 100 | 150.1275 | 150.373 | 91.040 | 20 | 30 |
| 2 | Metamphetamine-d5 (IS) |  |  | 155.1589 | 154.970 | 92.040 | 20 | 30 |
| 3 | Carbamazepine | 2.77 | - | 237.1019 | 237.304 | 194.097 | 19 | 57 |
| 4 | Carbamazepine-d10 (IS) |  |  | 247.1646 | 247.106 | 204.146 | 21 | 65 |
| 5 | Salbutamol | 0.44 | 100 | 240.1591 | 240.071 | 148.071 | 18 | 41 |
| 6 | Propranolol | 3.03 | 100 | 260.1642 | 260.070 | 116.111 | 18 | 62 |
| 7 | Propanolol-d7 (IS) |  |  | 267.2081 | 267.137 | 116.111 | 19 | 61 |
| 8 | Clenbuterol | 2.94 | 0.2 | 277.0865 | 276.971 | 202.995 | 16 | 60 |
| 9 | Clenbuterol-d9 (IS) |  |  | 286.1431 | 286.092 | 204.015 | 17 | 48 |
| 10 | Diazepam | 2.63 | 5 | 285.0785 | 284.995 | 193.054 | 32 | 82 |
| 11 | Diazepam-d5 (IS) |  |  | 290.1098 | 290.090 | 198.111 | 32 | 86 |
| 12 | Morphine | 0.99 | 50 | 286.1438 | 286.049 | 152.06 | 55 | 79 |
| 13 | Morphine-d6 (IS) |  |  | 292.1068 | 292.076 | 152.06 | 55 | 86 |
| 14 | Codeine | 1.20 | 2 | 300.159 | 300.385 | 165.054 | 39 | 78 |
| 15 | Codeine-d3 (IS) |  |  | 303.1778 | 303.122 | 165.071 | 41 | 82 |
| 16 | Cocaine | 1.97 | 100 | 304.1543 | 304.089 | 182.093 | 18 | 60 |
| 17 | Cocaine-d3 (IS) |  |  | 307.1726 | 307.055 | 185.111 | 20 | 50 |
| 18 | Sertraline | 5.06 | ≤300 | 306.0807 | 306.356 | 159.000 | 26 | 51 |
| 19 | Sertraline-d3 (IS) |  |  | 309.0994 | 309.030 | 158.929 | 31 | 106 |
| 20 | Methadone | 4.14 | 50 | 310.2161 | 310.048 | 265.007 | 15 | 57 |
| 21 | Methadone-d3 (IS) |  |  | 313.2347 | 313.272 | 268.166 | 15 | 57 |
| 22 | Oxycodone | 1.04 | 50 | 316.1539 | 316.098 | 241.054 | 27 | 71 |
| 23 | Lorazepam | 2.98 | 50-240 | 321.0188 | 321.054 | 274.889 | 21 | 72 |
| 24 | Citalopram | 3.58 | ≤300 | 325.1706 | 325.094 | 109.071 | 28 | 73 |
| 25 | Citalopram-d6 (IS) |  |  | 331.2081 | 331.119 | 109.071 | 28 | 92 |
| 26 | Bisoprolol | 2.30 | 100 | 326.2321 | 326.035 | 116.200 | 17 | 66 |
| 27 | Stanozolol |  | 2 | 329.2583 | 329.192 | 81.037 | 43 | 116 |
| 28 | Fentanyl | 4.12 | 1 | 337.2269 | 337.468 | 188.183 | 22 | 70 |
| 29 | Fentanyl-d5 (IS) |  |  | 342.2582 | 342.261 | 188.111 | 24 | 72 |
| 30 | Buprenorphine | 4.53 | 5 | 468.3102 | 468.250 | 396.111 | 38 | 119 |
| 31 | Buprenorphine-d4 (IS) |  |  | 472.3353 | 472.336 | 400.093 | 39 | 147 |

**Table S2** Figures of merit for determination of multiple substances in PBS spots via CBS-MS/MS. Red color denotes compounds that did not match the required MRPL levels, or showed poor accuracy/precision.

| **Compound** | **LOQ, ng·mL^-1^** | **Accuracy, %** | | | **Precision, %** | | |
| --- | --- | --- | --- | --- | --- | --- | --- |
|  |  | **3 ng·mL^-1^** | **40 ng·mL^-1^ ng·mL1^1^** | **80 ng·mL^-1^ L** | **3 ng·mL^-1^** | **40 ng·mL^-1^** | **80 ng·mL^-1^** |
| Methamphetamine | 0.5 | 93 | 103 | 98.3 | 3 | 2.1 | 2.8 |
| Carbamazepine | 1 | 107.7 | 102.6 | 97.8 | 9.1 | 1.8 | 1.8 |
| Propranolol | 1 | 96.5 | 101.8 | 97.7 | 7.0 | 2.2 | 1.4 |
| Clenbuterol | 1 | 102.4 | 104.9 | 97.6 | 6.4 | 1.5 | 1.2 |
| Diazepam | 0.5 | 102.2 | 101.1 | 97.1 | 5.8 | 5 | 2.2 |
| Codeine | 0.5 | 104.8 | 103.9 | 96.1 | 20.8 | 19.6 | 5.6 |
| Cocaine | 0.5 | 94.1 | 108.1 | 97.8 | 0.9 | 9.9 | 2 |
| Sertraline | 0.5 | 95.1 | 102.3 | 98.5 | 5.4 | 4.5 | 1.5 |
| Citalopram | 1 | 97.9 | 106.4 | 97.3 | 3.4 | 5.7 | 2.3 |
| Fentanyl | 0.25 | 95.1 | 110.3 | 100.6 | 1.6 | 1.6 | 2 |
| Buprenorphine | 0.25 | 102.1 | 101.8 | 95.4 | 3.7 | 7.4 | 3 |
| Morphine | 1 | 108.3 | 102.7 | 96.6 | 9.3 | 1.9 | 2.2 |
| Methadone | 0.25 | 95.5 | 106.8 | 97.5 | 1.9 | 2.7 | 1.2 |
| Salbutamol* | 5 | 120.3 | 133.3 | 110.5 | 37.5 | 25.2 | 14.4 |
| Oxycodone | 1 | 109.1 | 105.4 | 103.5 | 2.8 | 7.8 | 4.7 |
| Lorazepam | 25 | - | 95.5 | 88.3 | - | 19 | 7.3 |
| Bisoprolol | 0.25 | 98.4 | 109.5 | 95.4 | 9.8 | 12.3 | 16.2 |

**Table S3** Figures of merit for determination of multiple substances in plasma spots via CBS-MS/MS. Red color denotes compounds that did not match the required MRPL levels, or showed poor accuracy/precision.

| **Compound** | **LOQ, ng·mL^-1^** | **Accuracy, %** | | | **Precision, %** | | |
| --- | --- | --- | --- | --- | --- | --- | --- |
|  |  | **3 ng·mL^-1^** | **40 ng·mL^-1^** | **80 ng·mL^-1^** | **3 ng·mL^-1^** | **40 ng·mL^-1^** | **80 ng·mL^-1^** |
| Methamphetamine | 2.5 | 108.5 | 98.7 | 100.9 | 4.3 | 2.7 | 3 |
| Carbamazepine | 1 | 103.1 | 101.4 | 106.8 | 8.7 | 1.5 | 4 |
| Propranolol | 2.5 | 110.4 | 97.6 | 106.7 | 1.5 | 3.4 | 3.4 |
| Clenbuterol | 2.5 | 113.7 | 97.2 | 104.6 | 7.7 | 1.6 | 2.3 |
| Diazepam | 2.5 | 113.5 | 97 | 102.9 | 5.9 | 1.2 | 2 |
| Codeine | 5 | - | 96.2 | 94.1 | - | 8.6 | 11 |
| Cocaine | 2.5 | 110.9 | 98.5 | 102.1 | 7.4 | 1.3 | 0.9 |
| Sertraline | 2.5 | 100.5 | 91.3 | 107.3 | 5.1 | 1.8 | 3.1 |
| Citalopram | 2.5 | 107.5 | 95.5 | 103.5 | 2.9 | 3.8 | 1.5 |
| Fentanyl | 2.5 | 107.7 | 98.2 | 104.5 | 4.5 | 0.6 | 2.9 |
| Buprenorphine | 2.5 | 92.7 | 102.9 | 101.4 | 15.2 | 7.5 | 7.9 |
| Morphine | 5 | - | 99.5 | 97.4 | - | 5.2 | 5.9 |
| Methadone | 5 | - | 96.9 | 102.8 | - | 1.8 | 0.8 |
| Salbutamol* | 2.5 | 111.9 | 106.5 | 104.7 | 8.6 | 7.2 | 15 |
| Oxycodone | 5 | - | 99.2 | 110.8 | - | 3.13 | 1.11 |
| Lorazepam | - | - | - | - | - | - | - |
| Bisoprolol | 1 | 96.1 | 97.6 | 98 | 6.8 | 1.7 | 5.2 |

**Table S4** Figures of merit for determination of multiple substances in blood spots via CBS-MS/MS. Red color denotes compounds that did not match the required MRPL levels, or showed poor accuracy/precision.

| **Compound** | **LOQ, ng·mL^-1^** | **Accuracy, %** | | | **Precision, %** | | |
| --- | --- | --- | --- | --- | --- | --- | --- |
|  |  | **3 ng·mL^-1^** | **40 ng·mL^-1^** | **80 ng·mL^-1^** | **3 ng·mL^-1^** | **40 ng·mL^-1^** | **80 ng·mL^-1^** |
| Methamphetamine | 10 | - | 88.6 | 123.8 | - | 4.1 | 1.3 |
| Carbamazepine | 1 | 94.8 | 89.8 | 115.5 | 4.2 | 0.5 | 1.2 |
| Propranolol | 5 | - | 90.5 | 95.2 | - | 1.2 | 1.8 |
| Clenbuterol | 1 | 105.7 | 93.8 | 112.6 | 12.3 | 2.3 | 1.1 |
| Diazepam | 2.5 | 113 | 94.3 | 111 | 13 | 3.9 | 1.2 |
| Codeine | 2.5 | 102.4 | 95.3 | 75.3 | 2.5 | 3.8 | 16 |
| Cocaine | 2.5 | 119.8 | 93.3 | 96.8 | 10.9 | 1 | 4 |
| Sertraline | 5 | - | 92.3 | 101.8 | - | 0.2 | 0.1 |
| Citalopram | 2.5 | 118.6 | 93.1 | 111.6 | 7.7 | 2.5 | 3.7 |
| Fentanyl | 2.5 | 112.7 | 91 | 110.9 | 3 | 1.1 | 4.4 |
| Buprenorphine | 1 | 96.1 | 87.8 | 100.6 | 4.2 | 6.0 | 13 |
| Morphine | 10 | - | 92.9 | 23.8* | - | 1.9 | 22 |
| Methadone | 1 | 104.2 | 92.8 | 105.9 | 8.3 | 1.1 | 5.5 |
| Salbutamol* | 10 | - | 98.4 | 23.1* | - | 1.6 | 11.4 |
| Oxycodone | 10 | - | 93 | 135.0* | - | 1.6 | 17.7 |
| Lorazepam | 10 | - | 95 | 68.04 | - | 4.1 | 8.1 |
| Bisoprolol | 1 | 93.7 | 94.8 | 49.2* | 22.8 | 6 | 4.1 |
